# 495. Enterovirus Genotype Specific Immune Response in Cerebrospinal Fluid of Infected Infants

**DOI:** 10.1093/ofid/ofac492.553

**Published:** 2022-12-15

**Authors:** Kayla Shore, Anjana Sasidharan, Brian R Lee, Wail M Hassan, Christopher J Harrison, Rangaraj Selvarangan

**Affiliations:** University of Kansas School of Medicine, Kansas City, Kansas; Childrens Mercy Hospital, Missouri, Kansas; Children's Mercy Kansas City, Kansas City, Missouri; University of Missouri Kansas City, Kansas City, Missouri; Children's Mercy - Kansas City, Kansas City, Missouri; Children's Mercy, Leawood, Kansas

## Abstract

**Background:**

Non-polio Enteroviruses (EV) are important neonatal CNS pathogens. Multiple EV genotypes have been detected in pediatric CSF, e.g. Echovirus (E) 6 and E30. CSF innate immune responses to EV genotypes remain poorly defined. Most data are from EV-A71 or E30 CNS infections and do not compare responses between these or other EV types. We sought to better define innate immune responses to EV genotypes in CSF.

**Methods:**

Salvaged standard of care CSF samples from ≤6 month olds (real time EV PCR(+) or EV PCR(-) controls) from Jan 2010 - Dec 2020 were tested in duplicate on a 21- cytokine bead panel (MilliporeSigma). EV positive samples were previously genotyped by sequencing the viral capsid gene. Cytokine levels calculated from the standard curve were compared by Kruskal-Wallis and post-hoc analysis (GraphPad Prism 8.4.3). Natural partitioning of participants was explored using principal component analysis and cluster analysis (IBM SPSS v27). The utility of cytokine signatures in predicting EV status was explored using discriminant analysis and ROC analysis (IBM SPSS v27).

**Results:**

Data from 72 CSF with E6 (N=16), E9 (N= 9), E18 (N=9), and E30 (N=21) showed significant differences among EV genotypes vs. controls for 20 cytokines (IL-17 was excluded). Significant differences in cytokine levels in EV CSF vs controls were seen: E6 for all 20 cytokines; E9 for Fractalkine, IP10, and MCP1; E18 for Fractalkine and MCP1; E30 19 cytokines (not GM-CSF). PCA revealed only minor overlap of controls and EV positives; EV types overlapped, except E30, differing most from E9 and E18 but overlapping E6. The most important type-differentiating cytokines by PCA were MCP1, Fractalkine, IL-8, and IL-10. Patterns in DA resembled PCA; controls clearly separated from EV CSF, E30 being the most distinct. Overall, the discriminant model correctly classified EV type or controls at a 63% rate - highest for controls (94.1%) and E30 (74.1%). In the DA model, the most important cytokines were IP-10, IL-2, IL-1Ra, and Fractalkine. Discriminant scores had the largest area under the curve (AUC), 0.990. Among cytokines, IFNa2 had the largest AUC, 0.987.

**Figure:**
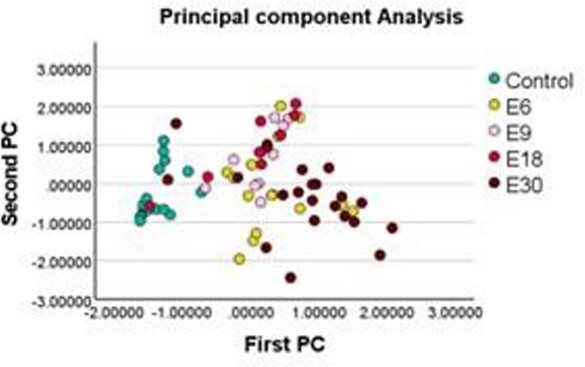

**Conclusion:**

Preliminary data show significant EV-genotype differences in innate immune response to CNS infections. Cytokine patterns may serve as a key predictor for discerning EV genotypes.

**Disclosures:**

**Brian R. Lee, PhD, MPH**, CDC: Grant/Research Support|Merck: Grant/Research Support **Christopher J Harrison, MD**, Astellas: Grant/Research Support|GSK: Grant/Research Support|Merck: Grant/Research Support|Pediatric news: Honoraria|Pfizer: Grant/Research Support **Rangaraj Selvarangan, BVSc, PhD, D(ABMM), FIDSA, F(AAM)**, BioFire: Grant/Research Support|Luminex: Grant/Research Support.

